# Solvent accessibility changes in a Na^+^-dependent C_4_-dicarboxylate transporter suggest differential substrate effects in a multistep mechanism

**DOI:** 10.1074/jbc.RA120.013894

**Published:** 2021-01-13

**Authors:** Connor D.D. Sampson, Matthew J. Stewart, Joseph A. Mindell, Christopher Mulligan

**Affiliations:** 1 School of Biosciences, University of Kent, Canterbury, Kent, United Kingdom; 2 Membrane Transport Biophysics Section, Porter Neuroscience Research Center, NINDS, National Institutes of Health, Bethesda, Maryland, USA

**Keywords:** anion transport, conformational change, membrane transport, protein conformation, protein chemical modification, transmembrane domain, transporter, tricarboxylic acid cycle, Vibrio cholerae, tricarboxylic acid cycle (TCA cycle) (Krebs cycle)

## Abstract

The divalent anion sodium symporter (DASS) family (SLC13) plays critical roles in metabolic homeostasis, influencing many processes, including fatty acid synthesis, insulin resistance, and adiposity. DASS transporters catalyze the Na^+^-driven concentrative uptake of Krebs cycle intermediates and sulfate into cells; disrupting their function can protect against age-related metabolic diseases and can extend lifespan. An inward-facing crystal structure and an outward-facing model of a bacterial DASS family member, VcINDY from *Vibrio cholerae*, predict an elevator-like transport mechanism involving a large rigid body movement of the substrate-binding site. How substrate binding influences the conformational state of VcINDY is currently unknown. Here, we probe the interaction between substrate binding and protein conformation by monitoring substrate-induced solvent accessibility changes of broadly distributed positions in VcINDY using a site-specific alkylation strategy. Our findings reveal that accessibility to all positions tested is modulated by the presence of substrates, with the majority becoming less accessible in the presence of saturating concentrations of both Na^+^ and succinate. We also observe separable effects of Na^+^ and succinate binding at several positions suggesting distinct effects of the two substrates. Furthermore, accessibility changes to a solely succinate-sensitive position suggests that substrate binding is a low-affinity, ordered process. Mapping these accessibility changes onto the structures of VcINDY suggests that Na^+^ binding drives the transporter into an as-yet-unidentified conformational state, involving rearrangement of the substrate-binding site–associated re-entrant hairpin loops. These findings provide insight into the mechanism of VcINDY, which is currently the only structurally characterized representative of the entire DASS family.

The divalent anion sodium symporter (DASS) family of transporters are present in all domains of life and are responsible for the transport of several key compounds into cells, such as citrate, Krebs cycle intermediates, and sulfate (1). Cytoplasmic citrate plays a major role in the metabolism of eukaryotic cells, contributing to the regulation of fatty acid, cholesterol, and low-density lipoprotein synthesis (2, 3, 4, 5). By maintaining and controlling the cytoplasmic citrate concentration, members of the DASS family (Transport Classification Database no. 2.A.47, SLC13 in humans) are key players in metabolic regulation in eukaryotes, as demonstrated by the phenotypes associated with their functional disruption. Knockdown of a DASS family member in fruit flies and nematodes leads to phenotypes analogous to caloric restriction, most notably a substantial increase in the lifespan of the organism, hence the alternative name for this family, INDY, which stands for “I'm not dead yet” (6, 7). In mice, knockout of a DASS family member (NaCT, SLC13A5 in humans) leads to protection against adiposity and insulin resistance (6), and knockout of the equivalent transporter in human hepatocarcinoma cells substantially reduces hepatoma cell proliferation and colony formation (7). Thus, DASS family members are prime targets for therapeutics designed to tackle metabolic diseases, including diabetes and obesity, and liver cancer.

DASS transporters are ion-coupled secondary active transporters. Secondary transporters can harness the energy stored in ion gradients (usually Na^+^ or H^+^) across the membrane to drive the energetically uphill movement of substrate against its concentration gradient. Secondary active transporters must occupy at least two major conformational states, the inward-facing state (IFS) and the outward-facing state (OFS), which alternate to expose the substrate-binding site from the cytoplasmic to the extracellular side of the membrane, and vice versa.

The DASS transporter family belongs to the ion transporter superfamily (8, 9), and the majority of characterized DASS transporters are known to transport their anionic substrates coupled to the co-transport of multiple Na^+^ ions (10, 11, 12, 13, 14, 15). However, some members of the DASS family are known to catalyze substrate exchange (*e.g.* the recently structurally characterized LaINDY, which is predicted to be an α-ketoglutarate/dicarboxylate exchanger) (16). The majority of our structural and mechanistic understanding of the DASS family comes from studies on a bacterial family member, VcINDY, from *Vibrio cholerae*, which is the only DASS co-transporter for which we have high-resolution structural information (17, 18). Functional characterization of VcINDY reveals that it preferentially transports C_4_-dicarboxylates (*e.g.* succinate, malate, and fumarate) (15). The model VcINDY substrate, succinate, is transported in its dianionic form coupled to the transport of three Na^+^ ions (15, 19). Our laboratory and others have revealed that VcINDY shares structural and functional characteristics with its mammalian homologues, suggesting that insight derived from the mechanism of VcINDY is directly applicable to the mammalian transporters (15, 19, 20).

The X-ray structures of VcINDY reveal that it forms a homodimer, and each protomer consists of two domains: the scaffold domain that forms dimer interface contacts, and the transport domain that houses key substrate-binding residues (Fig. 1*A*) (16, 17, 18). All structures of VcINDY to date are captured in the same IFS conformation, where the substrate is exposed to the cytoplasmic side of the membrane, suggesting that the crystallization conditions select for this lowest-free-energy state conformation of VcINDY (16, 17, 18). Using a combination of symmetry-based structural modeling and extensive biochemical and biophysical validation, we have described an OFS conformation of VcINDY in which the transport domain and the substrate-binding site are vertically translocated through the membrane (Fig. 1*A*) (21). Combined, the IFS structure and OFS model predict that VcINDY employs an elevator-like mechanism to achieve alternating access to the substrate-binding site from both sides of the membrane. Recent structural characterization of the DASS exchanger, LaINDY, revealed an OFS structurally very similar to the OFS predicted for VcINDY, strongly supporting the elevator-like mechanism for VcINDY and the DASS family in general (16).

**Figure 1:**
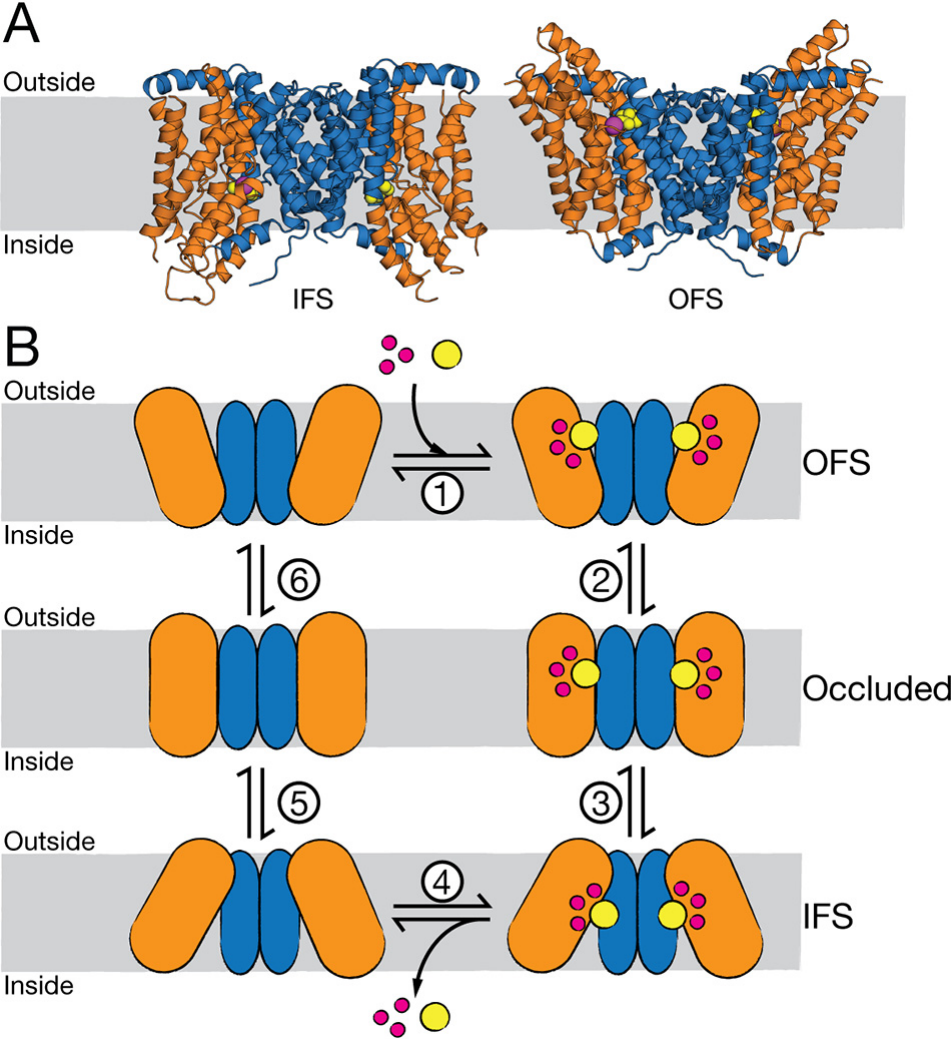
**Structure and simple kinetic transport scheme of VcINDY.***A*, IFS (*left*) crystal structure (PDB entry 4F35) and OFS (*right*) model of VcINDY. The transport domain is depicted as *orange helices*, and the scaffold domain is *blue*. Bound substrate is *yellow spheres*; the bound Na^+^ ion is a *magenta ball*; membrane is indicated by the *gray rectangle*. *B*, simple kinetic model of transport by VcINDY. Substrate-free OFS (*top left*) binds Na^+^ and succinate^2−^ in an unknown order (*step 1*), at which point the transporter transitions from OFS to IFS via an occluded state (*steps 2* and *3*). Substrates are released into the inside of the cell (*step 4*), and the empty transporter undergoes a conformational change back to the substrate-free OFS (*steps 5* and *6*). The *color scheme* is the same as in *A*.

The structures of VcINDY reveal that the substrate-binding site within the transport domain is composed of backbone and side-chain contacts from the tips of two re-entrant hairpin loops and an unwound helix (TM 7) (17). This organization is reminiscent of other elevator-like ion-driven transporters where local conformational changes of hairpin loops are required for ligand binding, controlling coupling ion and substrate access to the binding site, and as a means of preventing uncoupled transport (22, 23, 24). However, the X-ray structures of VcINDY reveal that, unlike these other elevator-like transporters, VcINDY's hairpin loops do not fully enclose the bound substrates; nor is there any structural evidence that they are involved in gating binding site access (17, 18). The transport of substrate and coupling ion is tightly coupled, meaning that Na^+^ transport cannot occur without succinate transport, and vice versa. In a simple kinetic scheme for VcINDY, succinate^2−^ and three Na^+^ ions bind to the OFS (Fig. 1*B*, *step 1*), facilitating a conformational change into the IFS via an intermediate occluded state that is yet to be structurally characterized (Fig. 1*B*, *steps 2* and *3*). The substrates are released from the IFS (Fig. 1*B*, *step 4*), and the empty transporter transitions back to the OFS via an occluded intermediate state (Fig. 1*B*, *steps 5* and *6*). The key to tightly coupled transport is that the IFS-to-OFS transition *cannot* occur unless the transporter either is ligand-free or carrying its full complement of substrate and coupling ions. What remains to be determined is how the presence of substrates influences the conformational state of VcINDY and whether local conformational changes of the re-entrant hairpins are required for transport.

Site-directed alkylation of single cysteine residues has been a valuable tool in elucidating the dynamic features of transporters, in particular the pioneering work on lactose permease by Kaback and co-workers (25, 26). Alkylation of single cysteine residues provides a readout of the accessibility of a particular position on the protein. The reactivity of single cysteine residues to hydrophilic thiol-reactive reagents depends on the solvent accessibility of the amino acid residue in a given conformation. Thus, any change in the reactivity between the cysteine and the thiol-reactive reagent reflects a change in the local environment and/or solvent accessibility to that particular position in the protein.

Here, we sought to explore the substrate dependence of the elevator-like conformational changes of VcINDY. To achieve this, we employed the hydrophilic, thiol-reactive reagent, methoxypolyethylene glycol maleimide 5K (mPEG5K) to probe the solvent accessibility of substituted single cysteines that are predicted to be accessible in the IFS or the OFS, but not both, according to the IFS crystal structure and OFS model. Our findings are consistent with VcINDY entering a structurally uncharacterized conformation upon binding substrates, with the majority of these accessibility changes being induced by binding Na^+^ alone. Several positions toward the tips of the hairpin loops were identified that, unlike all other positions tested, displayed substantial sensitivity to the binding of succinate over and above the sensitivity to Na^+^ binding. Taken together, these observations are consistent with a transport mechanism that involves multiple conformational changes that differ in their substrate dependence. In addition, our findings suggest that Na^+^ must bind prior to succinate and that dicarboxylate substrates appear to bind with a surprisingly low affinity.

## Results

### Experimental approach and generation of substituted cysteine panel

**Figure 2:**
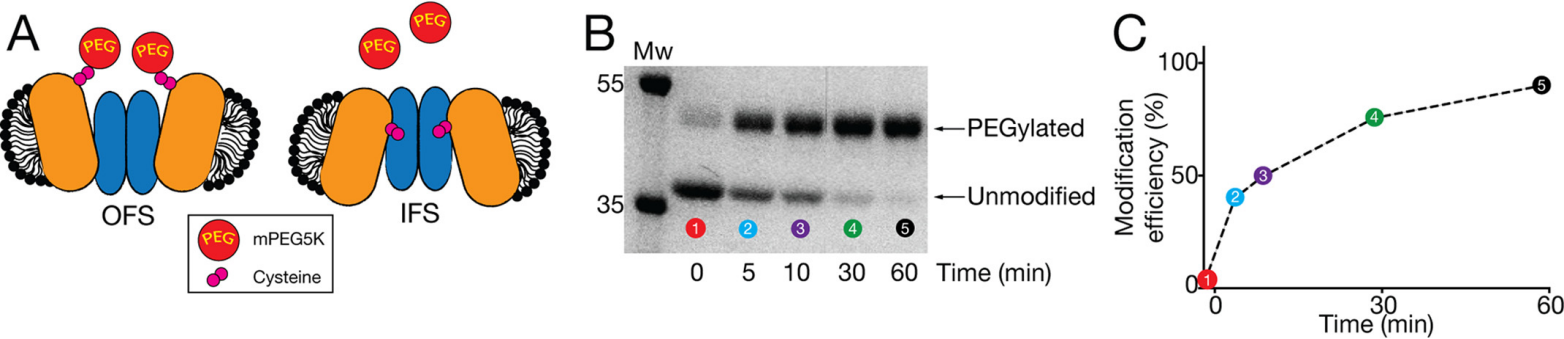
**Probing solvent accessibility of substituted cysteines.***A*, *cartoon representation* of VcINDY in its OFS (*left*) and IFS (*right*) depicting the change in solvent accessibility of a single substituted cysteine (*pink spheres*) in these different conformations. Cysteines that are more solvent-exposed will react more readily with the mPEG5K (*red spheres*), resulting in an increased rate of PEGylation. VcINDY's *color scheme* is the same as Fig. 1. *B*, an SDS-polyacrylamide gel depicting the gradual modification of a single cysteine residue of VcINDY by mPEG5K. Upon modification with mPEG5K, the apparent molecular mass of VcINDY increases from ∼38 to 43 kDa. Band intensity is quantified by densitometry. *C*, graph of the modification efficiency of the VcINDY cysteine mutant as a function of time using data derived from the SDS-polyacrylamide gel shown in *B*.

In this work, we sought to determine whether the presence of substrates drives VcINDY predominantly into its IFS, its OFS, or an intermediate conformational state that has not yet been structurally characterized. To probe the conformational state of VcINDY, we devised a substituted cysteine solvent accessibility assay in which we could measure the rate of modification of substituted cysteine residues using a hydrophilic cysteine reactive mass tag, mPEG5K, in the presence and absence of substrates (Fig. 2*A*). The reaction between an accessible single cysteine residue and mPEG5K would result in a 5-kDa increase in protein mass that is discernible on SDS-PAGE due to the PEGylated protein's retarded electrophoretic mobility (Fig. 2*B*). Digitization and densitometric analysis of the distribution of bands in each sample allows us to quantify the extent of PEGylation and calculate a modification efficiency for each time point (Fig. 2*C*). Plotting the modification efficiency for each sample in a time course provides us with a means of monitoring the modification rate of a particular position on the protein, which reflects the relative solvent accessibility of that position.

To allow us to differentiate between the IFS and OFS, we selected residues predicted to be accessible in the IFS or the OFS, but not both, using the IFS crystal structure and OFS repeat-swapped model as guides (17, 21). While avoiding highly conserved residues or residues involved in substrate or cation binding, we selected 24 amino acids in VcINDY to be individually substituted for cysteine, using a functionally active cysteine-free version of VcINDY as a background (see Table S1 for a full list of mutants tested) (15). Of the 24 single-cysteine variants produced, we discarded those mutants that were not produced in sufficient quantities for analysis, were incapable of catalyzing Na^+^-driven succinate transport, or were not reactive to mPEG5K under any conditions (Table S1). Following this sieving step, we were left with eight single-cysteine mutants (VcINDYA120C^OFS^, VcINDYT215C^OFS^, VcINDYS381C^OFS^, VcINDYL384C^OFS^, and VcINDYV388C^OFS^, which are predicted to be more accessible in the OFS (superior “OFS” denoting that they are predicted to be OFS-accessible cysteines), and VcINDYT154C^IFS^, VcINDYM157C^IFS^, and VcINDYT177C^IFS^, which are predicted to be more accessible in the IFS (superior “IFS” denoting their predicted IFS accessibility)) and a control cysteine mutant, VcINDYE42C, which is predicted to be equally accessible in both IFS and OFS. Importantly, all of the single-cysteine mutants used in this analysis were capable of catalyzing Na^+^-driven succinate transport, demonstrating that they can sample conformations essential for transport (Fig. S1). Using this panel of substituted cysteine mutants and the experimental approach described above, we sought to determine whether the presence or absence of substrates (Na^+^ and succinate) drives VcINDY into the IFS, the OFS, or a hitherto unidentified intermediate.

### Accessibility to all sites is substantially reduced in the presence of saturating substrate concentrations

**Figure 3:**
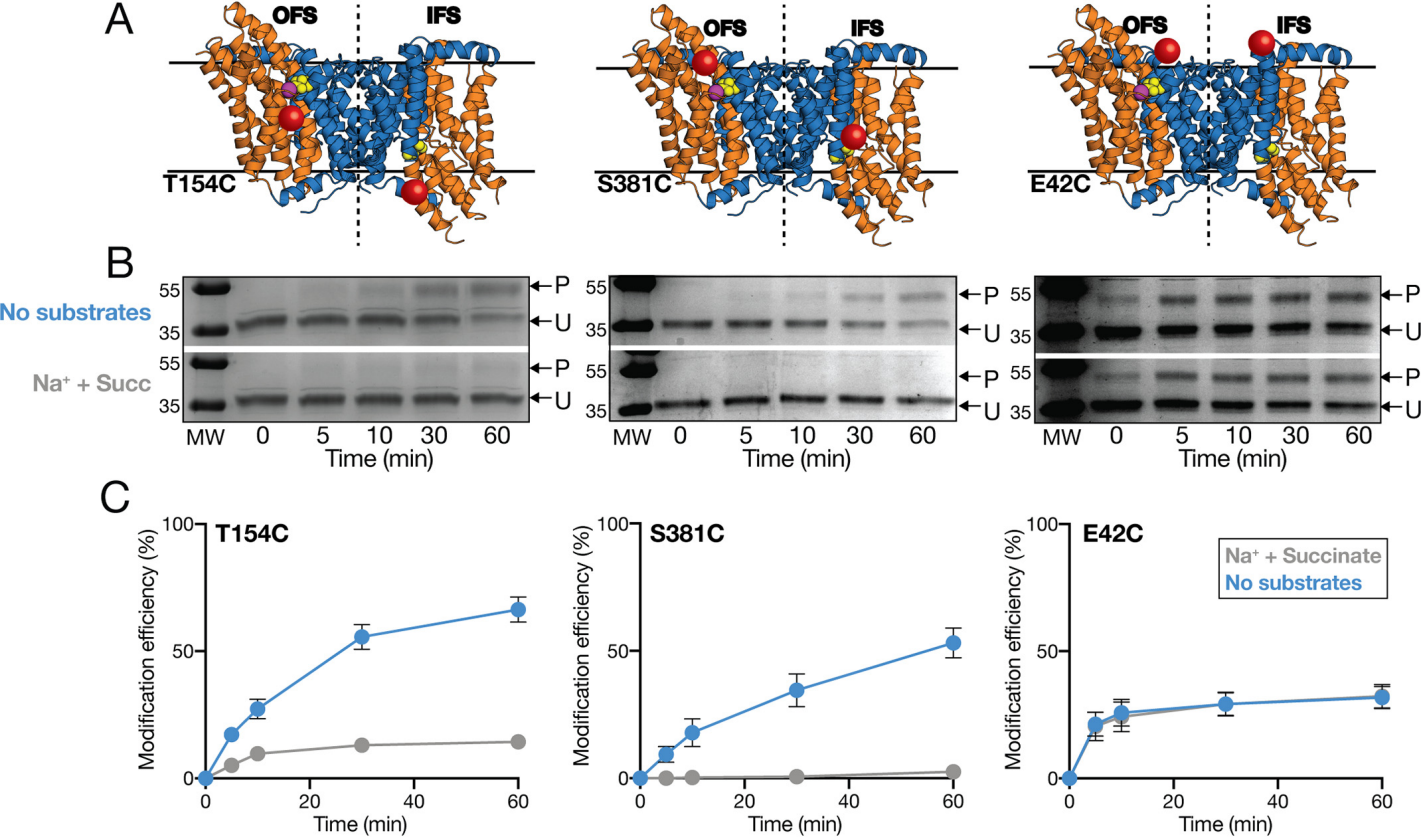
**Substrate-induced accessibility changes in VcINDY.***A*, a merge of the structures of the OFS (*left* of the *dotted line*) and the IFS (*right* of the *dotted line*), illustrating the relative accessibility of the single substituted cysteine residues (*red spheres*); T154C (*left*), S381C (*middle*), and E42C (*right*) in each conformation. The *color scheme* used for VcINDY is the same as in Fig. 1. *B*, representative SDS-polyacrylamide gels of PEGylation time course of the single cysteine mutants T154C (*left*), S381C (*middle*), and E42C (*right*) in the presence (*bottom gel*) and absence of substrates (*top gel*). The PEGylated protein bands (*P*) and unmodified protein bands (*U*) are indicated by *arrows*. The raw gel image data for each mutant displayed in *B* are re-used from Fig. S2A to allow comparison with the processed data in *C*. The *left panel* of *B* is from Fig. S2A (*i*), the *middle panel* of *B* is from Fig. S2A (*viii*), and the *right panel* of *B* is from Fig. S2A (*ix*). *C*, proportion (%) of each single cysteine mutant modified at each time point in the presence (*gray data*) of saturating Na^+^ and succinate and in apo conditions (*blue data*). Data points are the average of triplicate data sets, and the *error bars* represent S.E. This experiment was performed on three separate occasions with the same result.

To provide a readout on the conformational state of VcINDY, we selected two mutants for initial analysis that, based on available structural information, we predict are only accessible in the IFS or the OFS: VcINDYT154C^IFS^ (Fig. 3*A*, *left*) and VcINDYS381C^OFS^ (Fig. 3*A*, *middle*), respectively. To assess substrate-induced changes to the modification efficiency of these positions, we incubated each mutant protein with saturating substrate concentrations (1 mm succinate, 150 mm NaCl) or under apo conditions (no succinate and with Na^+^ ions replaced with experimentally inert K^+^) and quantified the rates of modification using SDS-PAGE and densitometric analysis (Fig. 3, *B* and *C*).

In the apo state, we observe substantial PEGylation of the IFS-accessible VcINDYT154C^IFS^ over the time course of the experiment, resulting in PEGylation of ∼65% of the protein (Fig. 3*C*, *left*, *blue data*, expressed as modification efficiency, which is acting as surrogate for modification rate). However, in the presence of saturating substrates, PEGylation of this position was almost completely prevented, suggesting that VcINDY favors a conformation in which Thr-154 is not solvent-accessible under these conditions (Fig. 3*C*, *left*, *gray data*).

Based on our current understanding of the structural mechanism of VcINDY, these data suggest that the presence of substrates drives VcINDY into a non-IFS conformation, which, based on our two-state model, is the OFS. We reasoned that if this change in accessibility of T154C is indeed due to a rigid body elevator-like movement of VcINDY's transport domain, then we should observe the opposite effect of substrates on a single cysteine in a position predicted to be accessible in the OFS, but not in the IFS. To test this hypothesis, we analyzed the PEGylation rate of the OFS-accessible cysteine variant VcINDYS381C^OFS^, in the presence and absence of substrates. In the absence of substrates, we observed a steady labeling rate of VcINDYS381C^OFS^ over the course of our experiment (Fig. 3 (*B* and *C*), *middle*). However, instead of seeing a further increase in the modification rate upon the addition of substrate, which would be consistent with our hypothesis, we observe that labeling of VcINDYS381C^OFS^ is almost completely blocked in the presence of Na^+^ and succinate (Fig. 3 (*B* and *C*), *middle*).

To rule out the possibility that the presence of substrate could be directly diminishing the reactivity of mPEG5K through some unforeseen mechanism, we performed our PEGylation assay on VcINDYE42C, whose single cysteine is predicted to be equally accessible in both of the known conformations of VcINDY (Fig. 3*A*, *right*). For this control mutant, we observed equal PEGylation rates in the presence and absence of substrates, indicating that the effect on the modification efficiency of our IFS- and OFS-accessible mutants is indeed due to interaction of the substrate with VcINDY (Fig. 3 (*B* and *C*), *right*).

**Figure 4:**
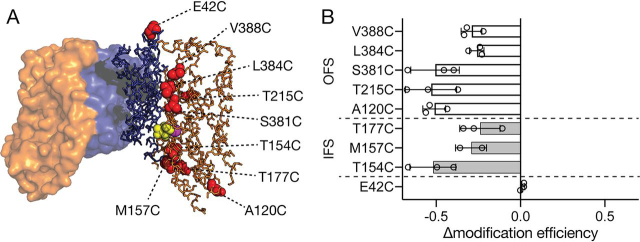
**Summary of the effects of the addition of substrates (Na^+^ and succinate) on the accessibility of all single cysteine mutants.***A*, IFS X-ray structure of VcINDY showing the positions of each single cysteine substitution used in this analysis. The *color scheme* is the same as in Fig. 1. Each of the positions mutated to cysteine is shown with a *red sphere*. *B*, change in modification efficiency (Δmodification efficiency) is the difference between the modification efficiency at the 60-min time point in the presence and absence of substrates. Using the value obtained in the absence of substrates as a baseline, a negative value indicates lower modification efficiency in the presence of substrate, and a positive value indicates higher substrate-induced modification efficiency. *Gray bars*, predicted IFS-accessible mutants; *open bars*, predicted OFS-accessible mutants; *E42C*, control. Individual data points are shown (*open circles*), each *bar* is an average of the individual data sets, and the *error bars* indicate S.D. This experiment was performed for each mutant at least three times.

Intriguingly, our data for VcINDYT154C^IFS^ and VcINDYS381C^OFS^ indicate that *both* positions become less accessible in the presence of substrates, which is consistent with VcINDY adopting an as-yet-unknown intermediate conformational state in the presence of substrates. To explore this possibility further, we analyzed the modification rates of the six other VcINDY variants with single cysteines positioned in sites spanning the scaffold domain-transport domain interface; two predicted to be only accessible in the IFS and four OFS-accessible only (Fig. 4*A*). Each of these six cysteine variants could be robustly PEGylated in the absence of substrates with final proportions of PEGylated protein ranging between 60 and 85%; these variations likely reflect the relative solvent accessibility of each position (Figs. 2*A* and 3). Whereas we also observed mutant-to-mutant variation in the magnitude of substrate-induced modification efficiency changes, *all* of the positions tested exhibited substantially reduced levels of modification in the presence of substrates compared with the absence, signified by a negative Δmodification efficiency value (Fig. 4*B*). These results indicate that all of the positions tested are less solvent-accessible in the presence of substrates, further suggesting that VcINDY favors a conformational state substantially different from the currently known conformations of VcINDY.

### Modification rates are influenced by the presence of Na^+^

To investigate the substrate-induced changes in modification efficiency in more detail, we sought to determine the individual effects of the coupling ion, Na^+^, and the substrate, succinate, on the modification efficiency of each substituted cysteine. To do this, we measured the PEGylation rate of our panel of single-cysteine mutants in one of four different conditions; Na^+^ alone; succinate alone; apo; or Na^+^ and succinate. In each case, we ensured that the reactions were osmotically and ionically balanced using KCl, which is known not to catalyze transport or interact specifically with VcINDY (15, 19).

**Figure 5:**
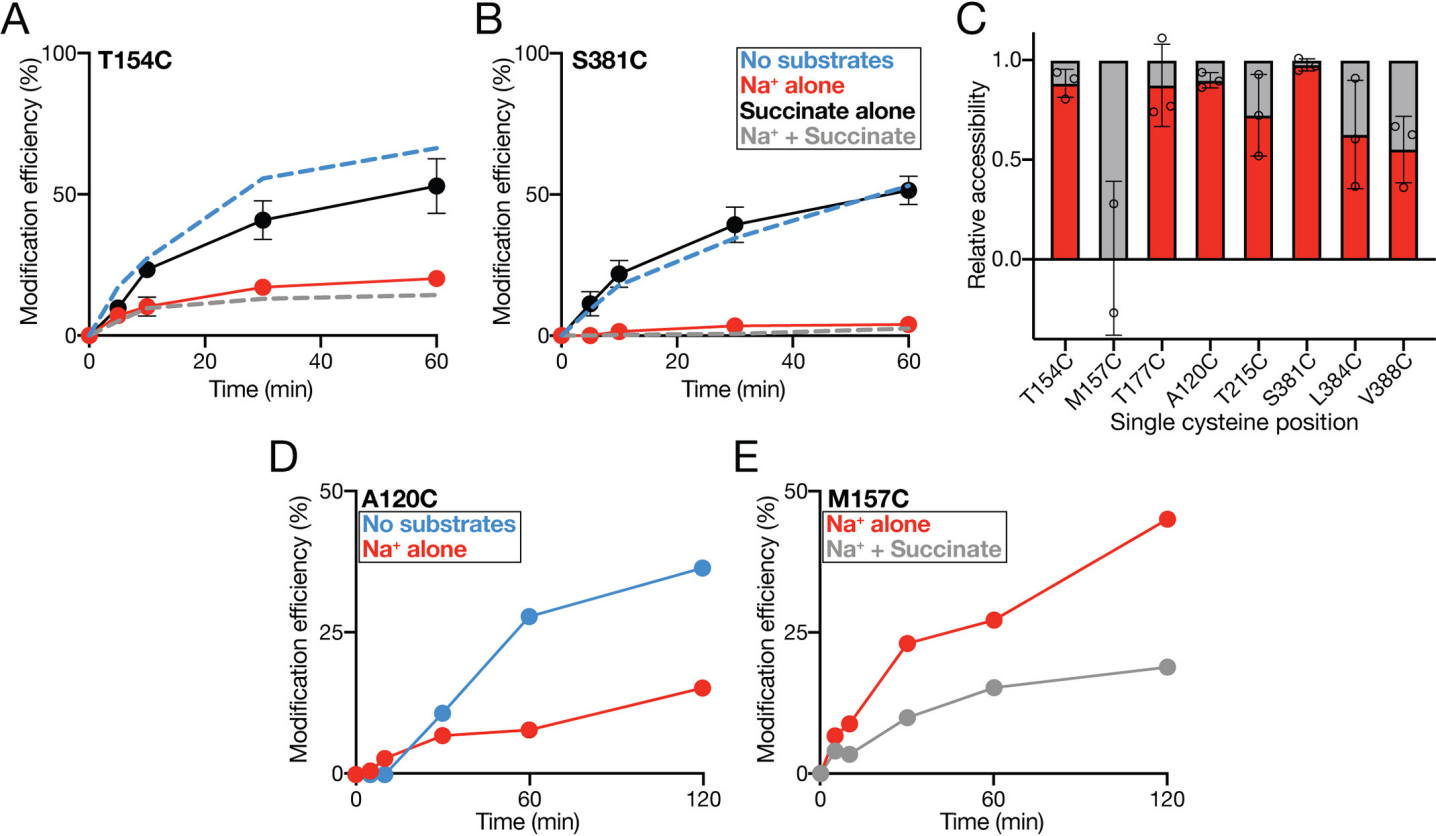
**Effects of individual substrates on the single cysteine modification efficiency.** Shown is the proportion (%) of VcINDYT154C (*A*) and VcINDYS381C (*B*) that is modified at each time point in the presence of Na^+^ alone (*red data*) or succinate alone (*black data*). The modification rate of each mutant in the presence and absence of saturating substrates (same data presented in Fig. 3) is shown as *dashed lines* for comparison (*gray* and *blue data*, respectively). The data shown are the average of three data sets, and *error bars* indicate S.E. *C*, summary of the effects on the modification rates for each mutant of Na^+^ alone (*red column*) compared with the overall effect of Na^+^ and succinate (*gray column*). The data are an average of two data sets for M157C and three data sets for all other mutants. Individual data points are shown (*open circles*), and *error bars* indicate S.D. This experiment was performed for each mutant at least three times. *D*, proportion (%) of VcINDYA120C in native nanodiscs modified in the presence of no substrate (K^+^-containing buffer, *blue data*) and Na^+^ alone (*red data*). *E*, proportion (%) of VcINDYM157C in native nanodiscs modified in the presence of Na^+^ alone (*red data*) and Na^+^ + succinate (*gray data*). Individual data sets are shown for experiments in *D* and *E*, which were performed on a single occasion.

We first analyzed the effects of coupling ion or substrate on the modification rates of the IFS- and OFS-accessible mutants, VcINDYT154C^IFS^ (Fig. 5*A*) and VcINDYS381C^OFS^ (Fig. 5*B*). For these two mutants, we observed no changes in the modification rate in the presence of succinate alone when compared with apo conditions (Fig. 5, *A* and *B*). However, the presence of Na^+^ alone substantially reduced the modification rate of these mutants, accounting for almost all of the reduction in the modification rate we observed in the presence of both Na^+^ and succinate (Fig. 5, *A* and *B*). These data suggest that the presence of Na^+^ alone is able to induce a shift in the protein's conformation that obscures these positions and reduces modification efficiency, whereas succinate binding by itself contributes minimally. In addition, the observation that succinate alone is unable to influence the modification rate of these positions indicates that one or more Na^+^ ions must bind to VcINDY prior to succinate binding. We next tested the other members of our single-cysteine panel to see whether Na^+^ binding also reduced their modification rate. In total, Na^+^ binding alone accounted for the majority of substrate-induced modification rate changes in half of the single-cysteine mutants, including two IFS-accessible mutants, VcINDY154C^IFS^ and VcINDY177C^IFS^, and two OFS-accessible mutants, VcINDYA120C^OFS^ and VcINDYS381C^OFS^ (Fig. 5*C* and Fig. S3).

In addition to Na^+^-induced modification rate changes, three members of our mutant panel, VcINDY215C^OFS^, VcINDY384C^OFS^, and VcINDY388C^OFS^, also exhibited further substantial modification rate reduction upon the addition of succinate in the presence of Na^+^. These additional succinate-induced modification rate changes strongly suggest that the sequential binding of each substrate stabilizes discrete conformations of VcINDY.

Interestingly, the amino acid positions that exhibit separable sensitivity to both Na^+^ and succinate are located on the arms of the re-entrant hairpin loops that contribute to binding site formation, raising the possibility that the observed accessibility changes are due to substrate-induced local conformational changes of the hairpin loops. Whereas this is the first evidence suggesting hairpin loop movement in VcINDY's mechanism, hairpin loops have been shown to perform a pivotal role in gating and coupling in other elevator-like transporters (23, 24, 25).

**Figure 6:**
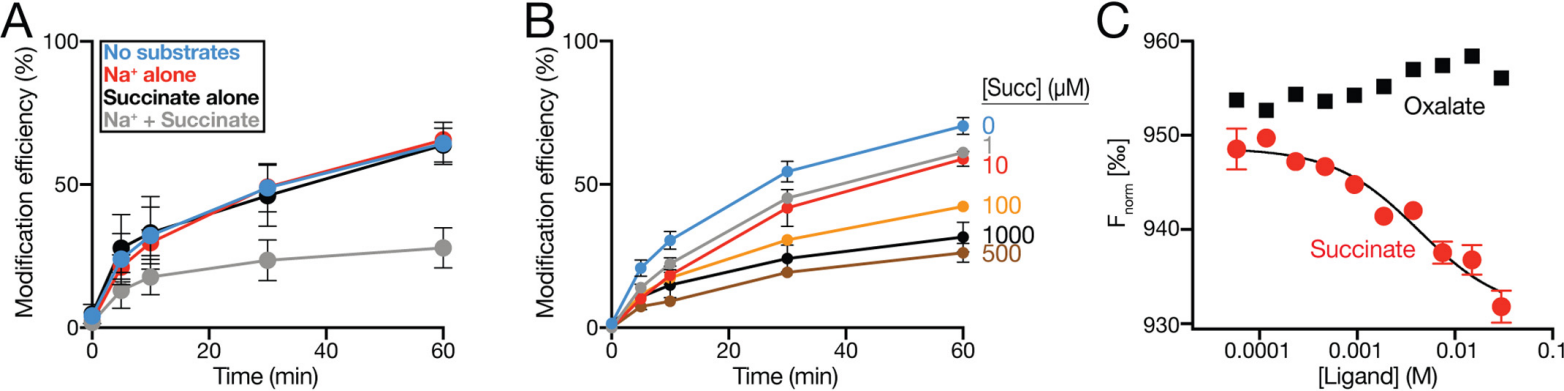
**Effects of substrates on the modification efficiency of VcINDYM157C^IFS^.***A*, modification efficiency (%) of VcINDYM157C^IFS^ at each time point in the presence of no substrate (150 mm K^+^, *blue data*), Na^+^ alone (*red data*), succinate alone (*black data*), and Na^+^ + succinate (*gray data*). *B*, modification efficiency of VcINDYM157C^IFS^ in the presence of 150 mm NaCl and increasing concentrations of succinate from 0 to 1 mm. The data are an average of at least three data sets for *A* and two data sets for *B*. *C*, MST-based binding analysis of VcINDY for succinate (*red data*) and nonsubstrate, oxalate (*black data*). Succinate data are an average of three data sets, and a single data set is shown for oxalate. *Error bars*, S.E. Each experiment was performed at least three times with the same outcome.

In contrast to all other positions tested, the modification efficiency of one of these re-entrant hairpin loop residues, VcINDYM157C^IFS^, was completely insensitive to the presence of Na^+^ ions alone but exhibited substantial succinate sensitivity in the presence of 150 mm Na^+^ (Figs. 5*C* and 6*A*). Succinate did not induce changes in the modification rate of VcINDYM157C^IFS^ in the absence of Na^+^, again suggesting an ordered binding process in which one or more Na^+^ must bind prior to succinate binding (Figs. 5*C* and 6*A*).

As these data were collected using detergent-solubilized protein, we wanted to determine whether we could observe the same substrate-dependent effects on accessibility in a lipid bilayer. To test this, we extracted two of the more intriguing mutants, VcINDYM157C^IFS^, which is highly sensitive to succinate, and VcINDYA120C^OFS^, which is highly sensitive to Na^+^, from the lipid bilayer using styrene maleic acid (SMA) co-polymer to generate native nanodiscs (27). Using our nanodisc-embedded mutants, we found that the substrate-induced modulation of the PEGylation rate in nanodiscs mirrored the observation in detergent; the presence of Na^+^ decreased the accessibility of VcINDYA120C^OFS^ (Fig. 5*D* and Fig. S2B), and the presence of succinate reduced the accessibility of VcINDYM157C^IFS^ (Fig. 5*E* and Fig. S2B). These data demonstrate that the effects of substrates on residue accessibility in a detergent environment is a good representation of the effects in a more physiological setting of the lipid bilayer.

Whereas our data are consistent with the different substrate conditions stabilizing particular conformations of VcINDY, an alternative possibility is that, in the presence of substrates, the fully loaded transporter undergoes rapid IFS-OFS interconversion—so rapid, perhaps, that the maleimide, which has a relatively slow rate of reaction, does not have sufficient time to react, which would result in apparent inaccessibility. To test this possibility, we performed our PEGylation assay on VcINDYM157C^IFS^ in the presence and absence of succinate using MTS-PEG5K, whose methanethiosulfonate (MTS) moiety has a considerably faster reaction rate than maleimides (28). Under these conditions, we observed the same decrease in modification efficiency in the presence of succinate compared with the absence (Fig. S4), indicating that our observations with mPEG5K reflect conformational stabilization rather than altered protein dynamics.

### PEGylation rates of VcINDYM157C^IFS^ suggest ordered low-affinity binding of succinate

Due to the maverick nature of the VcINDYM157C^IFS^ mutant, we investigated this variant in more detail. To determine whether the apparent succinate-induced decrease in modification efficiency was due to a specific interaction between VcINDY and succinate, we performed our PEGylation assay on VcINDYM157C^IFS^ in the presence of increasing concentrations of succinate ranging from no succinate up to 1 mm, while keeping a constant Na^+^ concentration of 150 mm (Fig. 6*B*).

We observed a dose-dependent decrease in modification efficiency of VcINDYM157C^IFS^ with increasing succinate concentration, indicating that this effect is indeed due to succinate binding (Fig. 6*B*). However, substantial reduction in the modification efficiency is only apparent when the protein is incubated with ≥100 μm succinate, suggesting an unexpectedly low affinity for succinate, considering the *K_m_* for transport is known to be 1 μm (15). This apparent low affinity is unexpected and, despite the M157C mutant retaining the ability to catalyze Na^+^-driven transport, we considered the possibility that mutating this position in the arm of this re-entrant loop could interfere with substrate interactions and cause this low-affinity interaction. To investigate this possibility, we determined the binding affinity of VcINDY WT using microscale thermophoresis (MST), which revealed a *K_d_* of 4.3 ± 1.5 mm for succinate in the presence of 150 mm Na^+^, whereas no binding was observed for the nonsubstrate oxalate (Fig. 6*C*). These data reveal that both VcINDY WT and the M157C mutant have a binding affinity in the same millimolar range, demonstrating that the cysteine substitution in this position did not dramatically affect substrate interactions.

**Figure 7:**
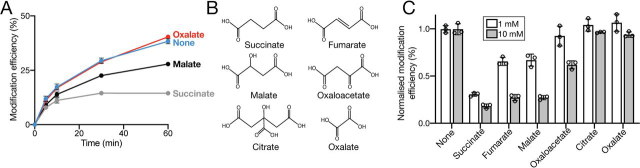
**Effects of substrates on the modification efficiency of VcINDYM157C^IFS^.***A*, proportion (%) of modified VcINDYM157C^IFS^ at each time point in the presence of 150 mm Na^+^ and no substrate (*blue data*), oxalate (*red data*), malate (*black data*), or succinate (*gray data*). *B*, chemical structures of the compounds used in *A* and *B*: succinate, fumarate, malate, oxaloacetate, citrate, and oxalate. *C*, normalized modification efficiency of VcINDYM157C^IFS^ after 1 h in the presence of 150 mm NaCl and either 1 mm (*open bars*) or 10 mm (*closed bars*) concentration of each indicated compound. The data are an average of at least three data sets, individual data points are shown (*open circles*), and the *error bars* indicate S.D. This experiment was performed at least three times.

In addition to succinate, VcINDY can transport a number of other C_4_-dicarboxylates, including malate, fumarate, and oxaloacetate, but not shorter dicarboxylates, such as oxalate, nor tricarboxylates, such as citrate (15). If the decrease in PEGylation rate is indeed due to VcINDY's specific interaction with its substrates, we would expect other known substrates to be similarly influential, whereas nontransported dicarboxylates should have no effect. Compared with Na^+^ alone conditions, substituting succinate with malate in the PEGylation reaction resulted in a significant increase in the modification rate, whereas substituting succinate with the nonsubstrate oxalate resulted in modification rates akin to those observed in the absence of any dicarboxylate substrate (Fig. 7*A*). These data indicate that the reduction in modification rate of VcINDYM157C^IFS^ is due to specific substrate interactions and not due to unforeseen indirect effects of dicarboxylates. However, we noted with interest that the presence of malate led to substantially and significantly reduced protection of the cysteine from PEGylation compared with succinate (Fig. 7*A*).

Intrigued by this result, we extended the compound range in our VcINDYM157C^IFS^ PEGylation assay to include the other known VcINDY substrates, fumarate and oxaloacetate, and another nonsubstrate citrate (Fig. 7*B*). To test the effects of our extended compound range on the VcINDYM157C^IFS^ PEGylation rate, we determined the modification efficiency after a 60-min incubation with mPEG5K and a 1 mm concentration of each test compound (Fig. 7*C*, *open bars*). As expected, the presence of succinate reduced the modification to the greatest extent, whereas oxalate, citrate, and, surprisingly, oxaloacetate induced negligible changes to the modification efficiency compared with the absence of substrates (Fig. 7*C*). The presence of 1 mm fumarate and malate reduced modification efficiency substantially, but only half as much as succinate (Fig. 7*C*). We reasoned that these variations in the extent of labeling in the presence of different substrates could merely reflect differences in the affinity for each of the different substrates. To test this possibility, we performed the same PEGylation assay, but with a final concentration of 10 mm of each compound (Fig. 7*C*, *closed bars*). In the presence of increased concentrations of each compound, we observed no change in the modification efficiency in the presence of either oxalate or citrate (Fig. 7*C*). Increasing the concentration of oxaloacetate to 10 mm resulted in significantly decreased modification efficiency compared with 1 mm, and whereas increasing the concentration of fumarate and malate to 10 mm led to further reduction in the modification efficiency, they were still significantly less effective than 10 mm succinate (Fig. 7*C*). These data suggest that the differences in the modification efficiency of VcINDYM157C^IFS^ in the presence of different substrates is due primarily to differences in VcINDY's binding affinity for each substrate. In addition, these results reveal that VcINDY's substrate-binding site is not saturated in the presence of 1 mm substrate, suggesting that under these experimental conditions, VcINDY has a remarkably low affinity for its substrates.

## Discussion

In this work, we have described the first foray into determining the substrate-dependent conformational changes of VcINDY, which is a structural and mechanistic representative of the DASS transporter family. Using a site-specific alkylation strategy on detergent-solubilized protein, we have demonstrated that the modification rate, which serves as a proxy for solvent accessibility, of several broadly distributed positions in VcINDY can be modulated by the presence of substrates. We demonstrate that the majority of these changes in modification efficiency can be induced by the presence of Na^+^ alone. However, we also observe substantial, separable effects of succinate binding on the modification efficiency of multiple positions, suggesting discrete effects of the two substrates. Furthermore, we have identified a position whose modification rate is insensitive to the presence of Na^+^ but highly sensitive to succinate. Further analysis of this variant indicates that substrate binding by VcINDY is an ordered process in which one or more Na^+^ ions must bind prior to the substrate, succinate. In addition, substrate titration with this succinate-sensitive variant reveals that it binds its substrate with a low affinity, a finding supported by binding analysis of WT VcINDY. Interestingly, the changes in solvent accessibility for the panel of mutants tested cannot be reconciled by a simple switch between the IFS structure and the OFS model. Therefore, our data are consistent with a more complex multistep transport mechanism that involves local conformational changes of the re-entrant hairpin loops and possibly the formation of an intermediate state, a model strongly supported by recent structures of DASS family members.

### VcINDY likely undergoes multiple large- and small-scale conformational changes during transport

There are now many examples of structurally characterized transporters that are predicted to employ an elevator-like transport mechanism (24, 29, 30, 31, 32, 33, 34, 35, 36, 37, 38), which has revealed common features among many of them, including distinct scaffold and transport domains, a broken transmembrane helix containing an intramembrane loop that contributes to the substrate-binding site, and two re-entrant hairpin loops that enter the membrane but do not fully span it. Whereas structural differences exist between the predicted elevator-type transporters, they are all predicted (or have been shown) to undergo a substantial vertical translocation of the transport domain, usually accompanied by a pronounced rotation of this domain, to expose the substrate-binding site to both sides of the membrane.

Our data suggest that multiple positions predicted to be solvent-accessible in either the IFS or the OFS, but not both, of VcINDY become less accessible in the presence of Na^+^ (Fig. 4), consistent with the stabilization of an as-yet-unidentified conformation of VcINDY that could represent an intermediate state. Cation-dependent conformational changes have been observed for multiple Na^+^-driven elevator-like transporters (39, 40, 41). By occupying an intermediate state between the IFS and OFS, many of the residues tested could be obscured, leading to reduced modification. No intermediate-state structure exists for VcINDY, so we cannot map the required conformational changes directly onto a structure of VcINDY. However, intermediate states of other elevator-like transporters, either cross-link–stabilized or captured in the presence of substrate (33, 42), have been structurally characterized, demonstrating that an intermediate state is well-occupied during the elevator-like mechanism. Indeed, in the case of the best-characterized elevator transporter, Glt_Ph_, the protein only transitions between the OFS and intermediate state, or between the IFS and intermediate state in the presence of Na^+^ alone (41). Only when both the cation and substrate are present can the protein fully transition between the IFS and OFS (via the intermediate state) (41). This blockage of the cation-only–bound state is crucial to tight coupling in secondary active transporters and prevents cation leak, which could prove catastrophic to the cell.

In both VcINDY and Glt_Ph_, the tips of these re-entrant loops form part of the substrate-binding site, coordinating both the coupling ion and substrate (17, 18, 43). However, a major mechanistic difference between VcINDY and Glt_Ph_ is the role of the re-entrant loops in gating and coupling. In Glt_Ph_ and other glutamate transporter homologues, the substrate and coupling ions are fully enclosed in the transport domain (23, 43, 44, 45). An OFS crystal structure of Glt_Ph_ in the presence of the bulky inhibitor dl-threo-β-benzyloxyaspartate revealed that the outermost re-entrant loop, HP2, could be propped open to allow substrate access to the binding site (23). The local and relatively subtle conformational changes of this hairpin are crucial to tight coupling of these transporters; the re-entrant loop is “open” in the absence of the full complement of substrate and coupling ions, which prevents premature translocation of the binding site that would lead to uncoupled transport. The symmetrically related re-entrant loop, HP1, was thought to play a similar role on the cytoplasmic face of the transporter. However, recent structural analysis of the human neutral amino acid transporter (ASCT2), which is structurally related to Glt_Ph_, revealed that HP2 is also required to open in the IFS, whereas HP1 remains static (24).

**Figure 8:**
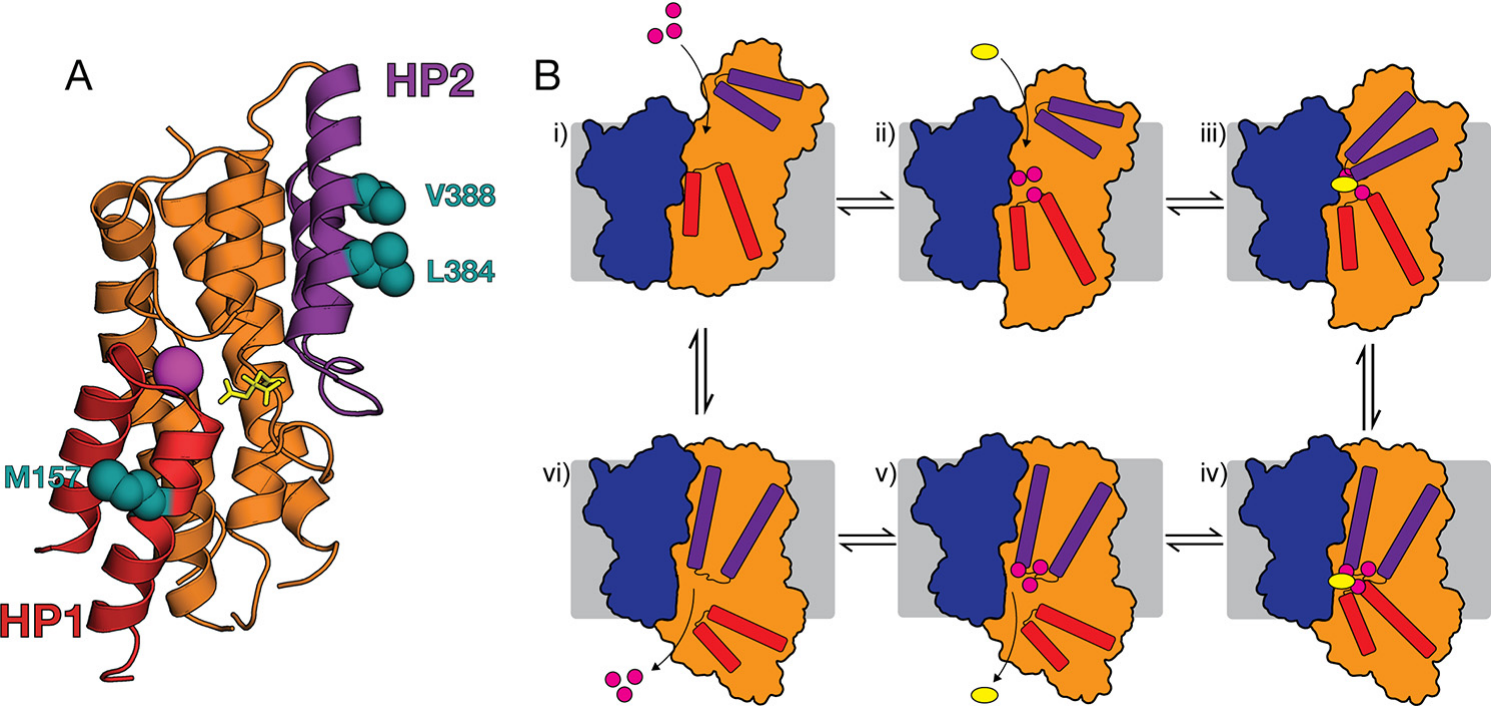
**Position of succinate-sensitive hairpin residues and a hypothetical mechanism of VcINDY.***A*, section of the X-ray structure of VcINDY's transport domain highlighting HP1 (*red*), HP2 (*purple*), and the three positions particularly sensitive to the presence of succinate (*teal*). The substrate (citrate, *yellow sticks*) and Na^+^ ion (*purple sphere*) are shown. *B*, hypothetical mechanism of VcINDY. OFS VcINDY binds Na^+^ ions (*i*), which stabilizes an open state of HP2 (*ii*); succinate binds (*iii*), which stabilizes the closed state of HP2 (*iv*), allowing the protein to transition to an inward-closed state (*v*); HP1 opens (*vi*); coupling ions and substrate are released into the cytoplasm (*v* and *vi*); empty transporter (*viii*) transitions back to the OFS to restart the cycle. The *color scheme* is the same as in *A*. Movement of hairpins and transport domains is represented by *color-matched arrows*.

In the IFS structure and OFS model of VcINDY, the substrate is solvent-exposed and straddles the interface of the scaffold and transport domains (17, 18, 21), potentially precluding the need for hairpin-coordinated gating in DASS transporters. In this study, we have probed the substrate-dependent accessibility of several residues in the substrate translocation pathway, including several positions in the re-entrant hairpin loops of VcINDY: Met-157 and Thr-154 in HP1 and Ser-381, Lue-384, and Val-388 in HP2 (Fig. 8*A*). Interestingly, we observe different effects of coupling ion and substrate binding within the same hairpin. As with other tested positions, Thr-154 and Ser-381 are mostly sensitive to the presence of Na^+^, whereas Leu-384, Val-388, and in particular Met-157 exhibit considerable sensitivity to the presence of succinate (Fig. 5*C*). Whereas further experimental work is required to provide a full picture of the dynamics of VcINDY's re-entrant hairpins, these results suggest discrete effects of the coupling ion and substrate on the conformation of the hairpins. This multistep mechanism is strongly supported by the recent cryo-EM structures of the Na^+^-bound IFS of VcINDY (16). Comparing these structures with the Na^+^- and succinate-bound IFS of VcINDY reveals substantial *local* structural changes centered around the re-entrant hairpin loop, HP1 (16). Whereas an apo state structure is required to determine the exact structural effects of Na^+^ binding, these new structures reveal that the binding of succinate induces conformational changes over and above what is induced by Na^+^ binding, supporting the multistep mechanism suggested by our alkylation data.

Analogous to the situation with Glt_Ph_ and its homologues, binding of Na^+^ is likely required for high-affinity binding of succinate, and binding of the succinate molecule is required to stabilize the “closed” conformation of the re-entrant loop, which allows the elevator-like conformational change to take place (23, 44, 45, 46, 47). The suggestion that the re-entrant hairpins undergo conformational changes in response to succinate binding is strengthened by the observations made during a series of solvent accessibility studies on the eukaryotic DASS transporter, NaDC1 (20, 48, 49). In concordance with our work presented here, the binding of Na^+^ and succinate had substantial and separable effects on the solvent accessibility of residues in the arm of re-entrant hairpin 2 in NaDC1 (in the same region and Ser-381, Leu-384, and Val-388 in VcINDY), suggestive of discrete conformational changes upon each Na^+^- and succinate-binding event (20, 48, 49). In contrast to our observations with VcINDY, the HP2 re-entrant loop residues become more accessible in the presence of Na^+^ alone, and then less accessible upon the addition of succinate (48). The differences between these two systems may be explained by the experimental set-up; NaDC1 was probed in the lipid bilayer, in the presence of gradients and a membrane potential, and in a background with 10 native cysteines present, whereas, in our study, each VcINDY variant was detergent-solubilized and contained only one cysteine residue. Cysteine accessibility studies on another bacterial DASS family member, SdcS from *Staphylococcus aureus*, revealed that Asp-329 (Phe-291 in VcINDY), which is predicted to be on the outward facade of the protein, is accessible in the presence of Na^+^, but not in the absence (50). However, the equivalent position of VcINDY was not tested in this study. Curiously, in the same SdcS study, Asn-108 (Asn-94 in VcINDY) was shown to be accessible from both the external and cytoplasmic sides of the membrane. However, in both the IFS crystal structure and the repeat-swapped model of VcINDY, there is dense protein blocking access to this site from the cytoplasmic side of the membrane (50). This perhaps suggests that the mechanism of SdcS is considerably different from the current structural model based on VcINDY or that the conformational changes during transport are *substantially* more extreme than currently thought. Nevertheless, these studies combined suggest that there are discrete conformational changes upon binding Na^+^ and succinate to DASS transporters. Indeed, solvent accessibility studies on NaDC1 prior to the elucidation of the structure of VcINDY revealed that the accessibility changes in HP2 were temperature-insensitive, indicative that the modulation in accessibility was not due a *large* conformational change, but perhaps due to blockade of these possible binding site residues by succinate (48). However, our current structural understanding of the DASS family reveals that these residues are in the arm of HP2 and do not form part of the binding site. Therefore, these observed accessibility changes of HP2 residues could be explained by subtle, local conformational changes of the hairpin in response to succinate binding. More structural information is required to fully realize the DASS transporter mechanism; in particular, a structure of the apo state of VcINDY will be especially illuminating. The substrate-dependent effects on the accessibility of HP2 residues for VcINDY and NaDC1 suggest that local hairpin movements are a feature of both transporters and are indicative of a shared transport mechanism. Indeed, the generation of homology models of NaDC3's IFS and NaDC1's IFS and OFS (based on the IFS structure and OFS model of VcINDY) reveals a shared overall architecture and shared Na^+^- and substrate-binding sites (20, 51). In addition, these models identified residues in NaDC1 that potentially form a discrete OFS-binding site that would be formed by the action of the proposed elevator mechanism; mutation of the residues diminished Na^+^ and succinate transport activity, supportive of a unified elevator mechanism for the DASS transport family. Furthermore, a recent study of human NaDC1 and NaCT identified a cluster of positively charged residues, conserved in the SLC13 family, that appear to strongly influence transport activity, with Arg-108 (Val-118 in VcINDY) proving to be indispensable for transport activity (52). These residues are predicted, based on the structures of VcINDY, to form a short helix on the cytoplasmic facade of the scaffold domain. Whereas these residues do not appear to contribute directly to binding site formation, they are hypothesized to interact with HP1 during the elevator-like transport cycle (52), perhaps playing a role in stabilizing a particular ligand-bound state. We note with interest that the equivalent helix in VcINDY contains multiple charged residues and that the cysteine accessibility assay data presented here demonstrate that accessibility to a residue in this helix (Ala-120) is highly sensitive to the presence of Na^+^. These data combined reveal a more complex picture of transport regulation in the DASS family that requires further investigation to fully realize.

### Substrate binding appears to be ordered and low-affinity

In this study, we have identified a position in the arm of HP1, Met-157, whose accessibility is insensitive to the addition of Na^+^ but undergoes substantial accessibility changes in the presence of succinate (Figs. 5*C* and 6*A*). Probing this mutant in more detail, we discovered that the accessibility effects were only induced by the addition of compounds known to be transportable by VcINDY (*e.g.* succinate, malate, and fumarate), demonstrating that specific binding was required (Fig. 7). In addition, we did not observe any changes in accessibility upon the addition of succinate in the absence of Na^+^, demonstrating that Na^+^ must bind first before succinate can bind, as has been suggested in previous studies on DASS transporters (53). This ordered binding is consistent with other Na^+^-driven elevator transporters. The archetypal elevator transporter, Glt_Ph_, transports aspartate coupled to the co-transport of three Na^+^ ions (54, 55), with two Na^+^ ions binding first to the apo transporter to “prime” the binding site before the aspartate and final Na^+^ bind (47, 56, 57), allowing the hairpin to close and the OFS-to-IFS transition to occur. Our data suggest a similar ordered binding mechanism for VcINDY. However, more detailed analysis of the coupling ion and substrate binding is required to illuminate this process further.

When titrating succinate and monitoring the dose-responsive change in modification of VcINDYM157C^IFS^, we only observed substantial shifts in the modification efficiency once the succinate concentration reached ∼100 μm (Fig. 6*B*). This concentration is surprisingly high, considering that VcINDY's *K_m_* for succinate transport in proteoliposomes in the presence of a similar Na^+^ concentration is 1 μm (15). To put this in the context of other elevator-like transporters, Glt_Ph_ also has *K_m_* for transport of ∼1 μm (58, 59) but has a *K_d_* of 100 nm (47). Because VcINDY and Glt_Ph_ have a similar mechanism and identical *K_m_* values, it is not unreasonable to predict that they would have similar *K_d_* values. However, if this were the case, we would expect VcINDY's binding site to be saturated at 100 μm; the fact that the we continue to observe decreases in the modification rate with increasing succinate concentration demonstrates that it is not. We confirmed separately, using MST, that WT VcINDY has a succinate *K_d_* in the millimolar range, revealing that our alkylation assay was a reasonable reporter of substrate affinity, and demonstrated that the observed low affinity was not caused by deleterious effects of the cysteine mutation on substrate interactions. Transporters with *K_m_* values lower than their *K_d_* are rarely observed. However, one notable example is CaiT, which has a *K_m_* of ∼80 μm and *K_d_* of ∼3 mm (60). CaiT's apparent low affinity is due to the protein containing more than one functionally significant substrate-binding site (60). Due to the positioning of the substrate at the interface of the scaffold and transport domains in the crystal structures of VcINDY, we predict that VcINDY also contains at least two disparate binding sites; functional studies of NaDC1 also support this hypothesis (20). In addition, a pharmacological study of human NaCT suggests multiple substrate-binding sites (61). Therefore, the apparent low affinity we observe in this study is likely a consequence of multiple substrate-binding sites and is potentially of mechanistic importance. Whereas the results presented in this study indicate that alkylation rate analysis can give a reasonable approximation of substrate affinity, it is important to consider that cysteine accessibility assays are not *precise* reporters of substrate affinity. First, cysteine reactions may not reach completion due to side reactions of cysteines (*e.g.* oxidation) that render a population unreactive (28). Second, the protein could contain multiple substrate-bound states that may not provide equal access to the cysteine being probed. The latter case is of particular significance for transporters, whose mechanisms are frequently highly dynamic and consist of multiple well-populated conformations (62, 63). Alternatively, this apparent low affinity may be due to VcINDY being in a detergent-solubilized state with further functional analysis in the presence of a lipid bilayer needed to resolve this issue. However, the fact the our purified VcINDY can specifically bind both Na^+^ and succinate, discriminating against structurally related nonsubstrates of VcINDY, which requires the formation of specific binding pockets, suggests that the binding sites are well-formed in these assay conditions.

We performed this study on detergent-solubilized protein to make the results directly comparable with the crystal structure of VcINDY, which was crystallized in its detergent-solubilized form. In addition, we selected our panel of single-cysteine mutants based on the crystal structure and repeat-swapped model of VcINDY to report on these particular conformations.

Using lipid bilayer mimetics, such as detergents, may produce results different from what is seen in a membrane environment, where there is an absence of lateral pressure, other membrane proteins, specific protein:lipid interactions and electrochemical gradients. Indeed, single-molecule FRET experiments have demonstrated that Glt_Ph_ has appreciably different dynamic behavior in detergent micelles *versus* lipid bilayer (41, 64). However, we have demonstrated using two of our VcINDY mutants, one with an IFS-accessible cysteine (M157C) and one with an OFS-accessible cysteine (A120C), that the effects of substrate on accessibility in detergent mirror the substrate effects in the lipid bilayer of native nanodiscs (Fig. 5). In addition, site-directed alkylation studies on other transport proteins have shown directly that substrate-induced alkylation rate changes are similar in detergent compared with those measured in lipid bilayer (65). These data combined give us confidence that the qualitative differences we observe in the presence and absence of substrates are mechanistically relevant. The elevator-like mechanism requires large-scale conformational changes, which are almost certainly affected by the lipid composition (both lipid headgroup and hydrocarbon chain length and saturation). The dynamics and overall transport rate of the only other well-characterized elevator-like transporter, Glt_Ph_, is influenced by the presence and composition of the lipid environment, and molecular dynamic simulations have recently shown that large-scale bilayer deformation can be induced by elevator-like transporter conformational changes (41, 64, 66, 67, 68, 69). Thus, aside from any specific protein:lipid interactions that may be influencing VcINDY's function, the physical properties (*e.g.* flexibility) of the bilayer, which are dictated by the lipid composition, will clearly have a strong effect on its mechanism. How the lipid environment and electrochemical gradients across bilayers influences protein dynamics and substrate-dependent conformational changes of VcINDY is of great interest and is the subject of ongoing studies in our laboratory

### Potential implications for the mechanism of VcINDY

Whereas further work is needed to fully realize the dynamics of VcINDY, our data are consistent with VcINDY having a complex mechanism that consists of multiple large-scale and local conformational changes as described by the following hypothetical mechanistic scheme (Fig. 8*B*). For simplicity, we will start this transport cycle with VcINDY in its OFS. However, as VcINDY is a secondary active transporter, it has the ability to work in the reverse direction, depending on the direction and magnitude of the electrochemical gradients. In the OFS conformation (Fig. 8*B*, *state i*), VcINDY is initially ligand-free, and the overall architecture resembles the structure described by the repeat-swapped model (21). One or more Na^+^ ions bind (for simplicity, we are showing three Na^+^ binding), stabilizing a conformation of the transport domain in which HP2 is open and the binding site is primed to bind succinate (Fig. 8*B*, *state ii*). Succinate binds in the primed site (Fig. 8*B*, *state iii*), which stabilizes the “closed” state of HP2 (Fig. 8*B*, *state iv*), allowing the transport domain to move into the closed IFS (Fig. 8*B*, *state v*). HP1 opens (Fig. 8*B*, *state vi*), releasing the substrates into the cytoplasm (Fig. 8*B*, *states vi* and *vii*). HP1 closes, and the ligand-free transport domain can translocate to form the OFS and restart the cycle.

Overall, this work reveals the existence of substrate-dependent conformations of VcINDY that are likely crucial to its tightly coupled Na^+^-driven transport mechanism and sheds light on an important facet of the DASS transporter mechanism that may have implications in the development of state-dependent inhibitors of this important transporter family.

## Experimental procedures

### Molecular biology and cysteine mutant generation

All single-cysteine variants were generated in a previously characterized cysteine-free background in which all three native cysteines had been substituted for serine (15). Substitutions were made with a QuikChange II site-directed mutagenesis kit (Agilent Technologies). Expression plasmids were fully sequenced to ensure that the desired codon substitution occurred and that no unwanted secondary mutations were introduced. Cysteine mutants were selected based on the IFS crystal structure of VcINDY (PDB entry 4F35) (17) and the OFS model that was based on this structure (21).

### Expression of VcINDY

Over the course of this work, we modified the expression protocol of VcINDY three times in an effort to maximize its yield, which led to an increase in yield from 0.2 to >5 mg/liter culture for WT VcINDY. Changes in expression levels of VcINDY did not affect the quality of the protein produced, the PEGylation efficiency for each variant, or the transport activity of each variant (data not shown). In all three protocols, VcINDY and its variants were expressed in-frame with an N-terminal decahistidine tag from a modified pET vector (70). VcINDY was initially expressed as described previously (15, 17). Briefly, BL21-AI (Invitrogen) cells harboring the expression vector were grown in lysogeny broth supplemented with 50 μg/ml kanamycin and incubated at 37 °C until it reached an *A*_600_ of 0.8. Cells were rapidly cooled in an ice bath for 20 min, at which point expression was induced by the addition of 10 μm IPTG and 6.6 mm l-arabinose. The cells were incubated at 19 °C and grown for ∼16 h before being harvested and resuspended in Lysis Buffer (50 mm Tris, pH 7.5, 200 mm NaCl, and 10% (v/v) glycerol). Following disappointing yields via the above method, we adopted the MemStar method described by Drew and co-workers (71). Briefly, in this protocol, Lemo21 (DE3) (New England Biolabs) cells harboring the expression plasmid were grown in PASM-5052 medium (72), which was supplemented with 50 μg/ml kanamycin and 25 μg/ml chloramphenicol and incubated at 37 °C until it reached an *A*_600_ of 0.5. At this point, expression was further induced by the addition of 0.4 mm IPTG, and the cells were incubated for ∼16 h at 25 °C before being harvested and resuspended in Lysis Buffer.

The following small modifications to this protocol increased the yield of VcINDY ∼10-fold. The PASM-5052 medium was exchanged for the MDA-5052, and the kanamycin concentration was increased to 100 μg/ml. These changes likely led to an increase in VcINDY yield because of better maintenance of the kanamycin-resistant expression plasmid in the Lemo21 (DE3) cells. Lemo21 (DE3) (and other BL21 derivatives) grow robustly in high levels of kanamycin in high-phosphate media, such as PASM-5052 (72), whereas, in MDA-5052 medium, which contains half the phosphate concentration, Lemo21 are again sensitive, making the antibiotic selection of expression plasmid-containing cells more effective.

### Purification of VcINDY

VcINDY was purified as detailed previously (15). Briefly, resuspended cells were lysed by sonication, the lysate was clarified by centrifugation at 20,000 × *g* for 20 min, and the membrane fraction was isolated by ultracentrifugation at 200,000 × *g* for 2 h. Membrane vesicles were resuspended in Purification Buffer (50 mm Tris, pH 8, 100 mm NaCl, 5% (v/v) glycerol). For the purification of cysteine-containing variants of VcINDY, the Purification Buffer was supplemented with 0.5 mm tris-(2-carboxyethyl)phosphine (TCEP) to keep the cysteines in a reduced state. 0.5 mm TCEP was added to all purification buffers. VcINDY was solubilized by incubating the vesicles with 19.6 mm *n*-dodecyl-β-d-maltopyranoside (Glycon) for 1 h at 4 °C. Nonsolubilized material was removed by ultracentrifugation, and the soluble fraction was incubated with Talon metal affinity resin (Takara Bio) for 16 h at 4 °C. Loosely bound contaminants were eluted from the resin, and the detergent was exchanged by two rounds of washing using Purification Buffer supplemented with 1.96 mm *n*-dodecyl-β-d-maltopyranoside and 10 mm imidazole, followed by Purification Buffer containing 5.4 mm *n*-decyl-β-d-maltopyranoside (DM; Glycon) and 20 mm imidazole. Protein was eluted by incubating the resin with Purification Buffer supplemented with 5.4 mm DM and 10 μg/ml trypsin for 1 h at 4 °C. The purified protein was concentrated and stored at −80 °C. SMA-extracted VcINDY was purified in the same way except the protein was extracted by incubation with 3% (w/v) 3:1 SMA at room temperature for 1 h, detergents were withheld from all buffers, and the protein-bound resin was washed with 5 mm imidazole. All cysteine-containing proteins were stored in the presence of TCEP to keep the cysteines reduced.

### Protein reconstitution

Protein was functionally reconstituted as detailed previously (21). 25–100 μg of DM-solubilized and purified protein was diluted to 2 ml in Reconstitution Buffer (25 mm Tris, pH 8, 100 mm NaCl, 5% glycerol, and 3% DM) and mixed with 400 μl of 20 mg/ml *Escherichia coli* polar lipids (Avanti Polar Lipids). This mixture of protein/lipid was incubated on ice for 10 min followed by rapid dilution into 65 ml of Inside Buffer (20 mm Tris, pH 7.5, 1 mm NaCl, 199 mm KCl, and 1 mm DTT). The resultant proteoliposomes were collected by ultracentrifugation, resuspended in Inside Buffer to a concentration of 8 mg/ml lipid, freeze-thawed three times, and stored at −80 °C.

### In vitro transport assays

For transport assays, the required amount of proteoliposomes were thawed, extruded 11 times through a 400-nm filter, collected by ultracentrifugation, and resuspended to a final concentration of 80 mg/ml lipid. Transport assays were performed by rapidly mixing the prepared proteoliposomes with Reaction Buffer (20 mm Tris, pH 7.5, 100 mm NaCl, 100 mm KCl, 1 μm valinomycin, and 1 μm [^3^H]succinate (American Radiolabeled Chemicals). At frequent time points, samples were collected from the transport reaction and quenched by the addition of ice-cold Quench Buffer (20 mm Tris, pH 7.5, 200 mm ChCl). Proteoliposomes and the accumulated [^3^H]succinate were collected by rapid filtration through 200-nm nitrocellulose filters (Millipore). The filters were washed with 3 ml of Quench Buffer, dissolved in FilterCount liquid scintillation mixture (PerkinElmer Life Sciences), and the accumulated [^3^H]succinate was counted using a Hidex 300SL Liquid Scintillation Counter.

### PEGylation time course

To perform the PEGylation time course, detergent-solubilized protein was thawed and buffer-exchanged using Zeba Spin Desalting Columns (Thermo Fisher Scientific) to remove the 0.5 mm TCEP and exchange the protein into PEGylation Buffer (50 mm Tris, pH 7, 5.4 mm DM, 5% (v/v) glycerol). For PEGylation of SMA-extracted protein, the same PEGylation Buffer was used, but without the DM. The substrate-free apo sample was formulated by mixing protein solution with 1 m KCl and PEGylation Buffer to generate a final protein concentration of 10 μm and a final KCl concentration of 150 mm. The “Na^+^ alone” sample was formulated in the same way except KCl was substituted for NaCl. The same approach was used for the “Na^+^ + succinate” samples except that succinate was added to a final concentration of 1 mm. For the “succinate alone” sample, protein was mixed with 150 mm KCl and 1 mm succinate. The protein/substrate mixtures were incubated for at 10 min at room temperature, at which point the PEGylation reaction was started by the addition of either 0.4 or 5 mm mPEG5K (Sigma–Aldrich) for detergent- and SMA-solubilized samples, respectively. Samples were collected at various time points, and the reaction was terminated by the addition of SDS-PAGE sample buffer containing 100 mm methyl methanethiosulfonate (Sigma–Aldrich). The PEGylation reaction samples were analyzed using nonreducing polyacrylamide gels, which were stained with Coomassie Brilliant Blue dye to visualize the protein.

### Densitometric analysis

The intensities of the bands corresponding to the unmodified and PEGylated VcINDY protein bands were quantified using ImageJ software (73, 74). For each time point, the modification efficiency was calculated using the following equation.

(Eq. 1) $$\mathrm{Modification efficiency}\left(\%\right)=\left(\frac{PEGylatedband}{Unmodifiedband+PEGylatedband}\right)\times 100$$

For the final data sets, replicates (*n* ≥ 3) of the modification efficiency for each time point were averaged, and the S.E. was calculated. Where indicated, statistical significance was examined using unpaired *t* tests.

### MST assay

For the MST assays, substrate stock solutions were produced by dissolving substrates in MST Assay Buffer (50 mm Tris, pH 7, 10% glycerol) and pH-adjusted to pH 7 with KOH. His-tagged VcINDY was labeled using the NanoTemper RED-tris-NTA dye and diluted to 500 nm in MST Assay Buffer containing 300 mm NaCl. Labeled VcINDY was mixed in 1:1 ratios with various substrate dilutions and incubated at room temperature for 5 min before loading into capillary tubes. MST was performed using the NanoTemper Monolith NT.115. Standard binding affinity protocols in the MO.Control software were used. Δ*F*_norm_ represents the proportion of the initial fluorescence remaining in the path of the laser at 4–5 s after heating begins. Δ*F*_norm_ values were fitted to a sigmoidal curve, and *K_d_* was estimated using GraphPad Prism software.

## Data availability

All data are contained within the article.
