# Supplementary material for: Solvent accessibility changes in a Na+-dependent C4-dicarboxylate transporter suggest differential substrate effects in a multistep mechanism
Source: J Biol Chem. 2021 Jan 13;295(52):18524–38. doi: 10.1074/jbc.RA120.013894 (PMC7939474; doi:10.1074/jbc.RA120.013894)
Supplement: Supplementary file 1 [file mmc1.pdf]

**Supporting information**

**Supplementary Table 1. List of VcINDY cysteine variants produced during the course of this work.** For each variant, it is indicated whether the protein could be stably expressed, whether it was active in *in vitro* transport assays, whether it was modifiable with mPEG5K, and if so, could this modification be modulated by substrate.

| **Cysteine mutant** | **Expressed?** | **Active?** | **Modifiable by mPEG5K?** | **Modulated by substrate?** |
|---|---|---|---|---|
| E42C | Yes | Yes | Yes | No |
| F79C | Yes | Yes | No | - |
| A120C | Yes | Yes | Yes | Yes |
| W148C | Yes | Yes | No | - |
| T154C | Yes | Yes | Yes | Yes |
| M157C | Yes | Yes | Yes | Yes |
| T177C | Yes | Yes | Yes | Yes |
| Y178C | Yes | Yes | No | - |
| A206C | No | - | - | - |
| A208C | Yes | Yes | No | - |
| S213C | No | - | - | - |
| T215C | Yes | Yes | Yes | Yes |
| V272C | Yes | Yes | No | - |
| A346C | Yes | Yes | No | - |
| V364C | Yes | Yes | No | - |
| V370C | Yes | No | - | - |
| F375C | Yes | No | - | - |
| S381C | Yes | Yes | Yes | Yes |
| A383C | Yes | No | - | - |
| A384C | Yes | Yes | Yes | Yes |
| V388C | Yes | Yes | Yes | Yes |
| T391C | No | - | - | - |
| G430C | Yes | Yes | No | - |
| S436C | Yes | Yes | No | - |

**Supplementary Figure 1. Transport activity of all 9 single cysteine variants.** Initial rates of [$^3$H]-succinate transport into proteoliposomes containing cysteine-free VcINDY (VcINDYcysless) or one of the 9 single cysteine variants, normalised to VcINDYcysless activity. Background levels of [$^3$H]-succinate accumulation were determined using a protein-free liposome control ("Empty"). Data points are the average of triplicate datasets, individual datapoints are shown (open circles) and the error bars represent SD. This experiment was performed at least twice for each mutant.

**Supplementary Figure 2. SDS-PAGE analysis of PEGylation rate of single cysteine variants. A)** Representative gels of the modification timecourse of each single cysteine variant solubilised in detergent tested under each substrate condition; no substrates (150 mM $K^+$), $Na^+$ alone, succinate alone (in 150 mM $K^+$), and $Na^+$ and succinate. The raw gel image data for mutants T154C (i), S381C (viii) and E42C (ix) are displayed in Fig S2A for comparison with the raw data from the other cysteine mutants in our panel. These data are re-used in Fig 3B to allow comparison with the processed data in panel Fig 3C. Fig S2A panel (i) is from Fig 3B left panel, Fig S2A panel (viii) is from Fig 3B middle panel, and Fig S2A panel (ix) is from Fig 3B right panel. **B)** SDS-PAGE gels of VcINDYA120C and VcINDYM157C in native nanodiscs tested in the presence of no substrate and $Na^+$ only, or $Na^+$ only and $Na^+$ + succinate, respectively.

**Supplementary Figure 3. Densitometric analysis of PEGylation rate of all 9 single cysteine variants.** Proportion of each single cysteine mutant modified at each timepoint under different substrate conditions; no substrate (blue data), succinate alone (black data), $Na^+$ alone (red data), and $Na^+$ and succinate (grey data). Data points are the average of triplicate datasets and the error bars represent SEM. This experiment was performed on at least 3 separate occasions for each mutant with the same result.

**Supplementary Figure 4. Substrate induced accessibility changes of VcINDYM157C$^{IFS}$ measured by modification by MTS-PEG5K. (A)** SDS-PAGE gel of the MTS-PEG5K modification timecourse of VcINDYM157C$^{IFS}$. The PEGylated protein bands (P) and unmodified protein bands (U) are indicated by arrows. **(B)** Densitometric analysis of (A). Proportion (%) of VcINDYM157C$^{IFS}$ modified at each timepoint in the presence of 150 mM $Na^+$ alone (red data) or in the presence of 150 mM $Na^+$ and 1 mM succinate (grey data).
